# Thermoforming 2D films into 3D electronics for high-performance, customizable tactile sensing

**DOI:** 10.1126/sciadv.adv0057

**Published:** 2025-05-14

**Authors:** Jungrak Choi, Chankyu Han, Donho Lee, Hyunjin Kim, Gihun Lee, Ji-Hwan Ha, Yongrok Jeong, Junseong Ahn, Hyunkyu Park, Hyeonseok Han, Seokjoo Cho, Jimin Gu, Inkyu Park

**Affiliations:** ^1^ Department of Mechanical Engineering, Korea Advanced Institute of Science and Technology (KAIST), 291 Daehak-ro, Yuseong-gu, Daejeon 34141, South Korea.; ^2^ Electronics and Telecommunications Research Institute (ETRI), Daejeon 34129, South Korea.; ^3^ Department of Mechanical Engineering, Hanbat National University, Yuseong-gu, Daejeon 34158, South Korea.; ^4^ School of Mechanical Engineering, Kyungpook National University, 80 Daehakro, Bukgu, Daegu 41566, South Korea.; ^5^ Department of Control and Instrumentation Engineering, Korea University, Sejong 30019 21 South Korea.; ^6^ Department of Mechanical Engineering, University of California, Berkeley, CA 94720, USA.; ^7^ KAIST Institute (KI) for the NanoCentury, Korea Advanced Institute of Science and Technology (KAIST), Daejeon 34141, South Korea.

## Abstract

The demand for tactile sensors in robotics, virtual reality, and health care highlights the need for high performance and customizability. Despite advances in vision-based technologies, tactile sensing remains crucial for precise interaction and subtle pressure detection. In this work, we present a design and fabrication method of customizable tactile sensors based on thermoformed three-dimensional electronics. This approach enables ultrawide modulus tunability (10 pascals to 1 megapascal) and superior mechanical properties, including negligible hysteresis and high creep resistance. These features allow the sensor to detect a broad spectrum of pressures, from acoustic waves to body weight, with high performance. The proposed sensors have high sensitivity (up to 5884 per kilopascal), high linearity (*R*^2^ = 0.999), low hysteresis (<0.5%), and fast response (0.1 milliseconds). We demonstrate applications in human-computer interaction and health care, showcasing their potential in various fields. This platform provides a scalable solution for fabricating versatile, high-performance tactile sensors.

## Teaser

Thermoformed 3D electronics enable ultrawide modulus tunability for high-performance and customizable tactile sensing.

## INTRODUCTION

Recently, the growing demands of Industry 4.0 have driven notable interest in customizable tactile sensors with high sensing performance. These sensors, which deliver high-quality data for machine learning applications and meet diverse design requirements, are increasingly sought after for various applications, including human-computer interaction (HCI) (*1*–*8*), health care monitoring (*9*–*13*), and robotics (*14*–*16*). These tactile sensors require high sensing performance (*17*–*20*) (e.g., high sensitivity, low hysteresis, fast response time, and long-term stability), high customizability [e.g., detection of a wide spectrum of target pressure levels (from acoustic wave to workout monitoring)], and ease of fabrication. To make sensors suitable for real-world applications, they must interface with analog-to-digital converters (ADCs) through optimized circuits. Therefore, sensors should be designed to adjust sensitivity according to target pressure requirements while ensuring low cost and compactness of the measuring circuit. Achieving both customizable sensitivity and compact, cost-effective design remains a substantial challenge (*21*).

**Fig. 1.**
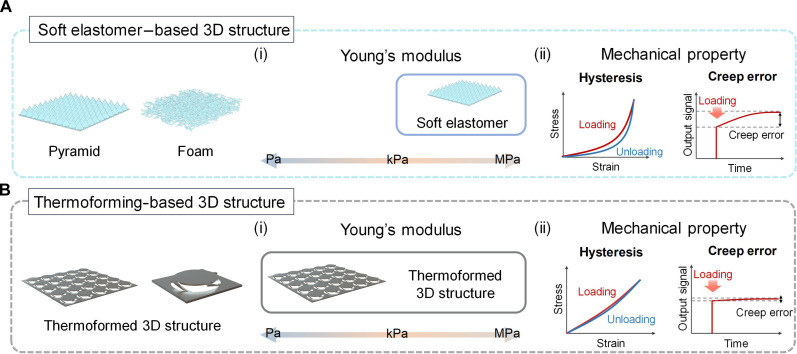
Comparative evaluation of soft elastomer–based 3D structure versus thermoforming-based 3D structure in terms of mechanical properties. Characterization of the soft elastomer–based 3D structure (**A**) and the thermoformed 3D structure (**B**) in terms of their Young’s modulus, illustrated on (i) a scale from pascals to megapascals and (ii) mechanical property graphs depicting hysteresis and creep error. This comparative representation highlights the enhanced mechanical stability and reliability of the thermoformed 3D structure for tactile sensor applications.

In general, the customizability and sensing performance of tactile sensors are mostly governed by the mechanical properties such as Young’s modulus, hysteresis, and creep and relaxation of the active layer. In particular, tuning the Young’s modulus of active layer enables to transmit different tactile feedbacks and acquire specific sensor response for target applications. Therefore, a wide modulus tunability is essential for sensor customization. Previously, soft elastomer–based customizable tactile sensors have been developed (Fig. 1A) (*21*–*26*). For example, microstructured soft elastomers have been developed using capacitive sensing mechanism and molding process. By designing geometries and controlling material parameters, the sensing performance was customized for various applications. Although this method could improve and customize sensing performance, the fabricated sensors still had several problems due to fundamental mechanical properties of soft elastomers such as high hysteresis and creep behavior. In general, soft elastomers such as Ecoflex (e.g., Ecoflex 00-30 and Ecoflex 00-50) or polydimethylsiloxane (PDMS; e.g., Sylgard 184 and Sylgard 527) exhibit viscoelastic behavior that restricts the sensors to have large signal drift, high hysteresis, and slow response time. Although several structuring and modification methods can reduce viscoelastic behavior, this reduced viscoelastic behavior is applied only under low-pressure range. These factors inhibit stable and accurate sensor responses when the sensor operates under various conditions such as long-term operation and time-varying pressure loading. Another method to fabricate tactile sensors is three-dimensional (3D) structuring of 2D rigid film that has advantages of high accuracy and long-term reliability (*27*–*29*). For example, compressive 3D buckling methods transform 2D flexible stiff films into 3D structured films. By designing the 2D film and controlling the elastomeric substrate, sensing performance can be customized. However, the sensor is prestressed and attached to the soft elastomer, which means that the elastomer is continuously under stress, negatively affecting sensing performance. Furthermore, the soft elastomer can dominate mechanical behavior, and the adhesion between soft materials and stiff polymer [e.g., polyimide or polyethylene terephthalate (PET)] is often poor. Consequently, it is still limited to widely tune the Young’s modulus of the combined materials, as the mechanical behavior is primarily influenced by the properties of the soft elastomer when the sensor is under pressure. In summary, it has been a considerable challenge to fabricate a tactile sensor with wide modulus tunability and high sensing performance simultaneously.

We, therefore, present a design and fabrication method of tactile sensors based on thermoformed 3D electronics (T3DE) of mechanically stiff polymer substrates with structural flexibility. T3DE, which is composed of a planar top surface and supporting legs, is fabricated by the thermoforming of patterned 2D electronics (2DE). In mechanical aspects, T3DE has excellent mechanical characteristics (negligible hysteresis and high creep resistance) and a wide modulus tunability (from ~10 Pa to ~1 MPa) (Fig. 1B). Here, the wide modulus tunability can not only customize sensing performance but also transmit tactile feedbacks when users touch them. In electrical aspects, we adopted a capacitive sensing mechanism with electrical shielding, which has advantages of low power consumption, low hysteresis, high repeatability, temperature insensitivity, and fast response. On the basis of these properties, the T3DE-based tactile sensor can be customized to detect a wide spectrum of target pressure levels, from acoustic wave sensing to workout monitoring, with high sensing performance. This includes high sensitivity (up to 5884 kPa^−1^), high linearity (*R*^2^ = 0.999), low creep error [<1% in 20 min at room temperature (25°C)], low hysteresis (<0.5%), fast response (0.1 ms, capable of detecting 10-kHz acoustic waves at ~60 dB), and excellent repeatability (<0.1% over 5000 cycles). These capabilities demonstrate a wide range of potential applications for tactile sensors in fields such as HCI and health care monitoring. We believe our platform will have a substantial impact on tactile sensing technology with its improved capabilities.

## RESULTS

### Overview of T3DE-based tactile sensor featuring high customizability and sensing performance

**Fig. 2.**
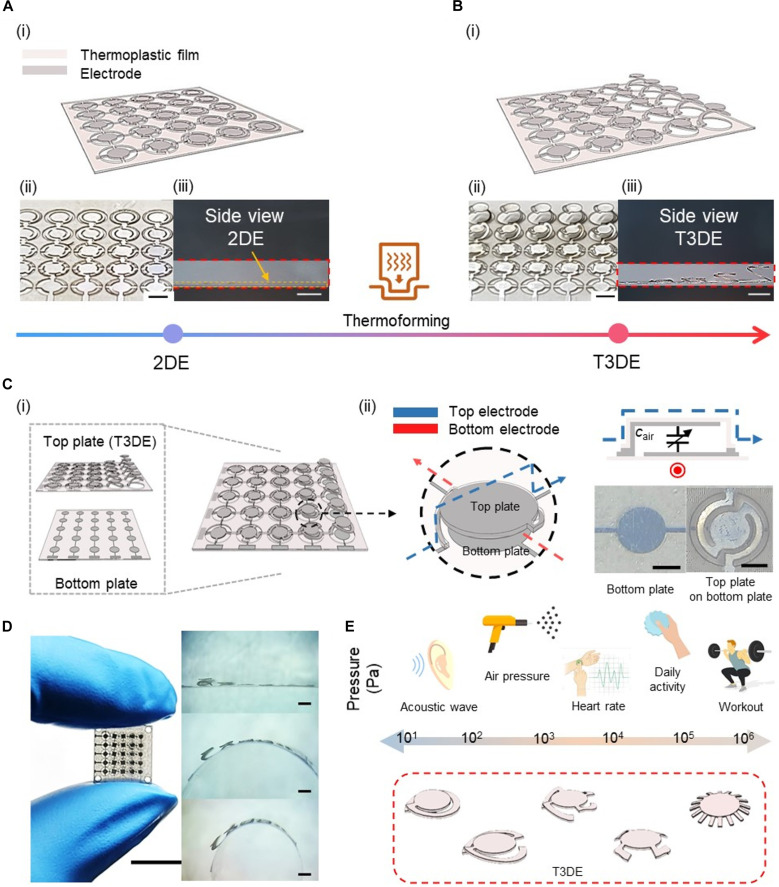
Overview of T3DE. The thermoforming process transforms patterned 2DE (**A**) into elastically deformable T3DE (**B**), depicted through (i) a schematic illustration, (ii) a bird’s eye view, and (iii) a side view. Scale bars, 2 mm. (**C**) The overview of the T3DE-based tactile sensor includes (i) a schematic illustration of the sensor and (ii) a depiction of the sensing mechanism, accompanied by an optical image of the bottom plate with the T3DE assembled on it. Scale bars, 1 mm. (**D**) Optical image of the fabricated T3DE-based tactile sensor (scale bar, 1 cm) along with its bending capability (scale bars, 2 mm). (**E**) Wide modulus tunability of T3DE, illustrating the Young’s modulus of biological materials and the tactile pressure range suitable for various applications. Photo credit: J. Choi, Electronics and Telecommunications Research Institute (ETRI).

Figure 2 presents an overview of the T3DE-based tactile sensor. The fabrication of the T3DE-based tactile sensors begins with the precise patterning of the electrode and thermoplastic film, a foundational step that sets the stage for the thermoforming process (Fig. 2A). The next step is the thermoforming process, which is a transformative step not only to convert 2DE into elastically deformable 3D configurations (Fig. 2B) but also to enable unprecedented customization of the sensor’s mechanical properties by adjusting the supporting legs’ design. With the capacitive sensing mechanism, leveraging a parallel plate capacitor with air as the dielectric offers distinct advantages in sensitivity and energy efficiency, underscoring the T3DE sensor’s superior performance. Then, the T3DE-based tactile sensors with a five-by-five array are fabricated by attaching the T3DE to a bottom plate (Fig. 2C-i) and incorporating a capacitive sensing mechanism based on a parallel plate capacitor with air as the dielectric medium (Fig. 2C-ii). The fabrication process is detailed in figs. S1 to S5. Our platform allows for T3DE-based tactile sensors as small as 2 mm in size and able to bend with small curvature radii (Fig. 2D). Figure 2E shows the wide tunability of Young’s modulus of T3DE, which is important for its sensing range and sensitivity. In general, the range of tactile pressure interactions for most applications falls between 1 Pa and 1 MPa (*30*). Compared to previously developed soft elastomer–based tactile sensors, the T3DE-based tactile sensor offers a wider range of modulus tunability, with the Young’s modulus capable of being tuned from 10 Pa to 1 MPa. This wide range of tunability enables the sensor to detect a broad range of pressure levels, from acoustic waves to body weight. In addition, the T3DE-based sensor is particularly useful for tactile feedback, as it can mimic the mechanical properties of many biological materials, which exhibit a broad range of Young’s moduli, from 10 Pa for fat tissue to 1 MPa for tendon (*31*). The benefits of the T3DE-based tactile sensor are further highlighted in the context of recent advancements in computational resources and machine learning, which have spotlighted the significance of sensor technology (*32*). To maximize the benefits of computational methods, the quality of data provided by the sensors must be of a high standard. However, many soft elastomer–based tactile sensors often face challenges such as hysteresis and creep error due to their viscoelastic properties, which can greatly affect the accuracy of the data obtained. In contrast, the T3DE-based tactile sensor is designed to operate within a small strain range in the elastic region, resulting in extremely small hysteresis and creep error. In addition, it uses air as the medium, making it less susceptible to environmental interference such as temperature and humidity. The T3DE-based sensor also has other advantages over soft elastomer–based tactile sensors, such as customizable pressure range, higher sensitivity, lower hysteresis, and faster response time, as will be discussed in the “Pressure sensing performance of T3DE-based tactile sensor” section.

### Modulus customization of T3DE

**Fig. 3.**
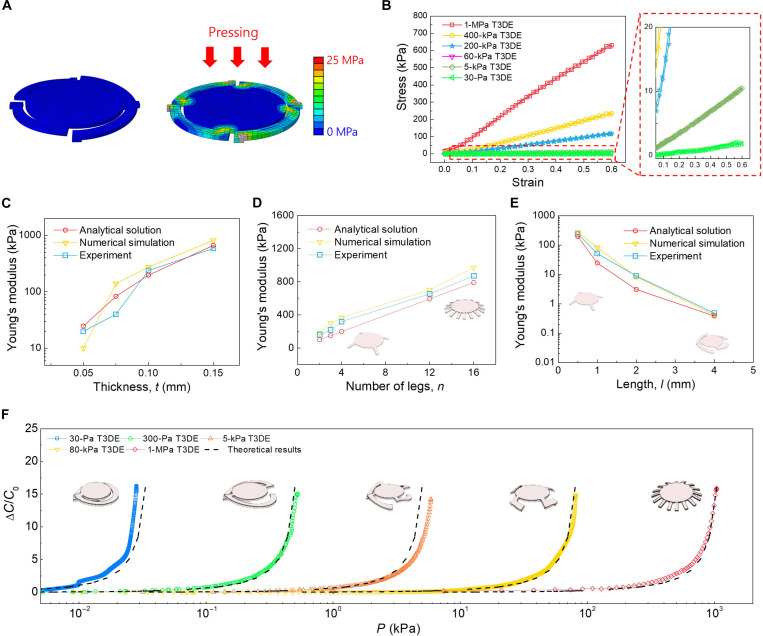
Numerical, analytical, and experimental analysis for predicting the Young’s modulus of T3DE and sensing performance of the T3DE-based tactile sensor. (**A**) Numerical simulation for compression stress analysis of the structure. (**B**) Experimental results of mechanical properties of the T3DE with different Young’s modulus of T3DE. (**C** to **E**) Effects of parameters such as thickness (C), number of legs (D), and length of legs (E) on the Young’s modulus of T3DE. (**F**) Normalized capacitance changes in response to pressure with experimental and theoretical results. There is a good match between the experimental and theoretical results.

The T3DE consists of the supporting legs and the top surface. The supporting legs determine the Young’s modulus, which can be customized through various designs. To predict the Young’s modulus of T3DE, we first built a numerical simulation model using the finite element method (FEM) to simulate the mechanical pressure response of the T3DEs (Fig. 3A). The simulation was conducted for thermoforming and device operation. Through thermoforming simulation, we optimized the geometry of the T3DE based on the maximum strain of the T3DE, which is less than the elastic limit of the electrode and the thermoplastic substrate during the thermoforming process and operation of the T3DE for stable electrical connection. Then, we evaluated the Young’s modulus of T3DE through the simulation of the device operation. To predict and observe the effects of geometry of the T3DE, an analytical approach is simpler and useful compared to FEM. To create the analytical model, we used the standard beam bending model to describe the behavior of the T3DE. This model is suitable owing to the minimal strain in the supporting legs, which facilitates reversible bending. Each leg of the T3DE can be considered as a beam. The governing equation for the stress-strain relation is obtained, as shown in Eq. 1

$$E_{T3\text{DE}}=\frac{P}{s}=\frac{F}{\mathrm{As}}=\frac{\text{Ewnh}t^{3}}{4{\mathrm{Al}}^{3}}$$ (1)

where F, *P*, *s*, *A*, *h*, *l*, *w*, *t*, *n*, *E*, and *E*_T3DE_ are compressive force, compressive pressure, compressive strain, area of top surface, height of the T3DE, length, width, thickness, number of legs, Young’s modulus of 2D plastic film, and Young’s modulus of the T3DE, respectively. Detailed explanation of the analytical solution is shown in fig. S1. For the experimental characterization of mechanical properties, a uniaxial compression test was conducted for the T3DE with different geometrical conditions. Figure 3B shows the empirical results of the mechanical properties of the T3DE with different Young’s moduli. Here, 1-MPa T3DE refers to the Young’s modulus of T3DE of 1 MPa. In particular, the T3DE exhibited elastic behavior when deformation remained within the elastic range of the original 2D plastic film, ensuring a stable correlation between electrode separation and applied pressure. This is particularly advantageous for capacitive sensors, as maintaining this correlation is essential for accurate pressure sensing. This results in low hysteresis and improved performance compared to conventional elastomer–based pressure sensors.

We investigated the effects of various parameters such as *t*, *n*, and *l*. The predicted Young’s modulus of the T3DE in the analytical solution was compared with the FEM and experimental results (Fig. 3, C to E). The key structural parameters optimized through the finite element analysis (FEA) simulations are summarized in table S1, where variations in leg dimensions and configurations were systematically adjusted to balance mechanical flexibility and stability. The good match between the analytical solution, simulation, and experimental results indicates the validity of the simulation model, with all three sets of values showing consistent trends. The results show that the Young’s modulus of thermoformed 3D structure can be precisely controlled and an ultrawide range of Young’s modulus can be obtained. The structural optimization process, including the FEA of various T3DE configurations, is illustrated in fig. S2, where the effects of leg length and the number of legs on mechanical response were analyzed to refine the sensor’s design. On the basis of various configurations of parameters, the Young’s modulus of T3DE can be precisely designed to have a target Young’s modulus in the range from ~10 Pa to ~1 MPa. On the basis of customization of the Young’s modulus, the sensing performance of T3DE-baesd tactile sensor can also be predicted and customized. A parallel plate capacitor model was used to evaluate and compare the normalized capacitance change $\left(∆C/C_{0}\right)$ in the T3DE-based tactile sensor, as shown in Eqs. 2 and 3

$$C\left(d\right)=\varepsilon_{0}\varepsilon_{\text{air}}\frac{A}{h}\;$$ (2)

$$\frac{∆C}{C_{0}}=\frac{C-C_{0}}{C_{0}}=\frac{1}{1-\frac{P}{E_{T3\text{DE}}}}-1$$ (3)

where $\varepsilon_{0}$ and $\varepsilon_{\text{air}}$ are vacuum permittivity and relative dielectric constant of air, respectively. Because $\varepsilon_{0}$, $\varepsilon_{\text{air}}$, and A are constant during the compression, the relative change of capacitance can be calculated by the distance between two electrodes, which is highly predictable compared to previously developed soft material–based sensors. Figure 3F confirms a good match between the experimental data and the calculated values of relative capacitance changes in response to the external pressures for capacitive-type tactile sensors. An *R*^2^ value of 97% confirms a good agreement between the two approaches for capacitance analysis. To complement the structural analysis, we conducted electrostatic simulations to analyze the electric field distribution and capacitance variations. The simulation results, presented in fig. S3, confirm that the predicted capacitance response aligns closely with theoretical calculations, validating the accuracy of our modeling approach. This customization capability enables the sensor to be finely tuned for a wide array of applications, from detecting the delicate nuances of acoustic waves to measuring the substantial force of body weight pressures.

### Pressure sensing performance of T3DE-based tactile sensor

**Fig. 4.**
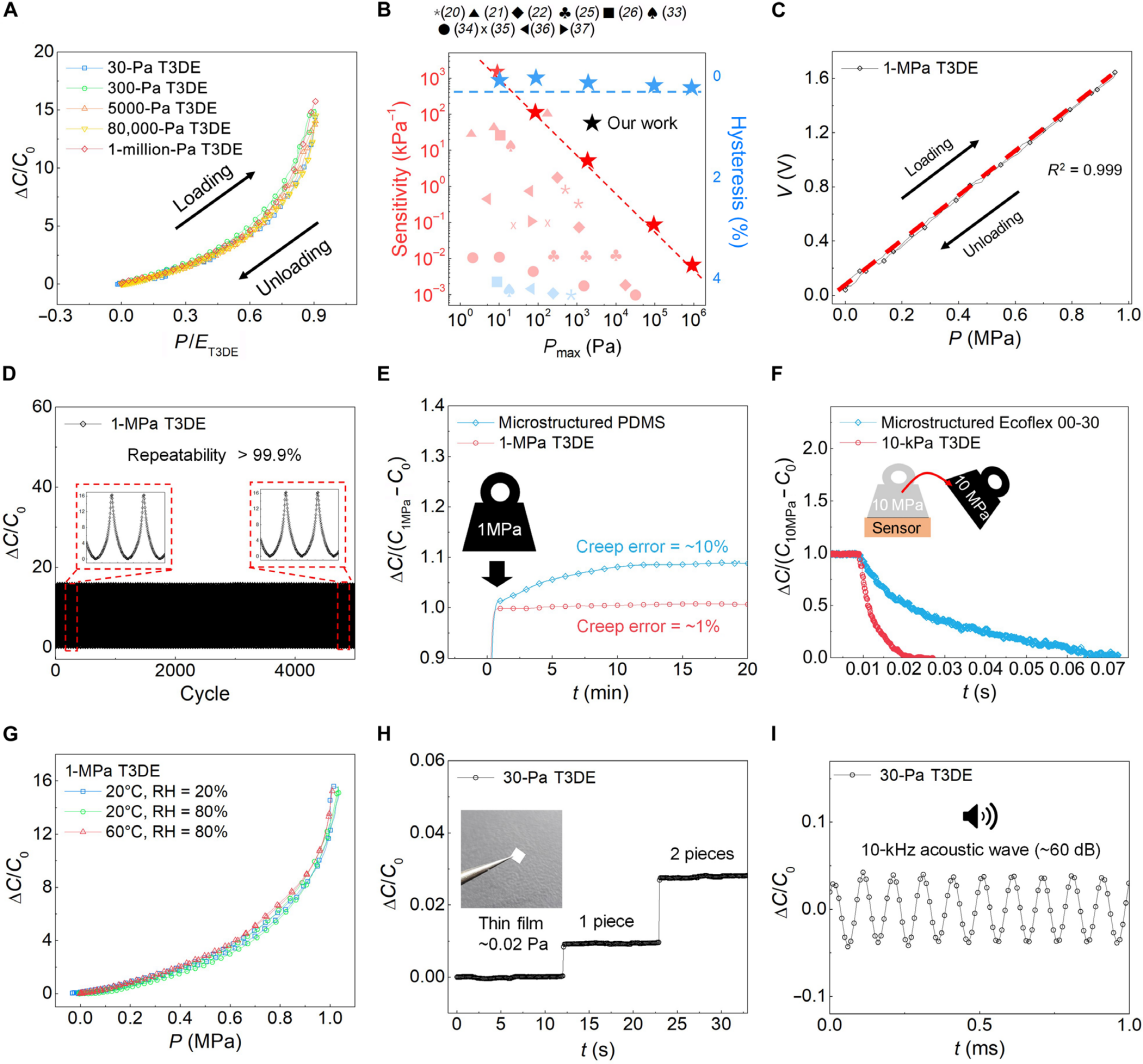
Pressure sensing performance of T3DE-based tactile sensor. (**A**) Normalized pressure sensing performance of each T3DE-based tactile sensor under loading and unloading. (**B**) Comparison of the sensitivity and hysteresis of the T3DE-based tactile sensor with state-of-the-art capacitive tactile sensors. T3DE-based tactile sensor outperforms contemporary sensors with higher sensitivity (up to ~6000 kPa^−1^) and lower hysteresis (less than 0.5%) over a wide pressure range. (**C**) Relationship between pressure (*P*) and voltage (*V*) using CDC under loading and unloading. The relationship shows high linearity (*R*^2^ > 0.999). (**D**) Repeatability test by applying 5000 repeated 1 MPa of compression/release cycles (full-scale operation). The repeatability was calculated to be greater than 99.9% during the 5000 cycles. (**E**) Creep error test compared to microstructured PDMS–based tactile sensor. Creep error of microstructured PDMS–based tactile sensor (~10%) is higher than the 1-MPa T3DE–based sensor (~1%). (**F**) Recovery performance compared to microstructrued Ecoflex 00-30–based tactile sensor. The 10-kPa T3DE is able to recover its output signal to its original state within only 0.02 s, which is much faster than the microstructrued Ecoflex 00-30–based tactile sensor. (**G**) Normalized pressure sensing performance of each T3DE-based tactile sensor under different temperature and humidity conditions. The sensor kept its performance up to 60°C and a relative humidity (RH) of 80%. (**H**) LOD of the sensor based on the 30-Pa T3DE, which was able to detect ultralow pressure without notable noise. (**I**) Dynamic response of the sensor based on the 30-Pa T3DE, which can detect 10-kHz acoustic waves with a response time of ~0.1 ms. Photo credit: J. Choi, Electronics and Telecommunications Research Institute (ETRI).

To apply customizable tactile sensors in various practical applications, it is essential to meet several requirements, such as high sensitivity, low hysteresis, fast response, and long-term stability. Figure 4A demonstrates the pressure sensing performance of T3DE-based tactile sensors, which not only meet these requirements but also outperform state-of-the-art tactile sensors. The nondimensionalized pressure sensing performance of each T3DE under loading and unloading, as shown by the relationship between *P*/*E*_T3DE_ and Δ*C*/*C*_0_, exhibits a similar tendency regardless of *E*_T3DE_, indicating that our customization method enables the pressure sensor to achieve consistent sensing performance for various T3DEs. In addition, each sensor exhibits less than 0.5% hysteresis, which is defined as the relative difference in the area underneath the Δ*C*/*C*_0_ versus pressure curves under loading and unloading cycles. The sensitivity of the T3DE-based tactile sensor is an important metric in evaluating its performance. It is defined as *S* = δ(Δ*C*/*C*_0_)/δ*P*. In the case of T3DE with the lowest Young’s modulus (30-Pa T3DE), the sensor exhibits a sensitivity up to 5884 kPa^−1^. To further assess the sensor’s performance under dynamic conditions, we conducted additional experiments to analyze cyclic capacitance changes under different force and frequency conditions (fig. S4). The results confirm that the sensor maintains stable performance across different pressure levels, showing no unexpected drift in capacitance or performance degradation. Figure 4B demonstrates that our T3DE-based tactile sensor outperforms contemporary sensors with higher sensitivity and lower hysteresis over a wide pressure range. To further assess the uniformity and performance distribution of the T3DE-based tactile sensor, we conducted additional statistical analyses. The results confirm that the T3DE sensor exhibits high uniformity, with performance variations remaining within 5% among different sensor positions, as shown in fig. S5. Specifically, our sensor shows superior sensitivity not only in the low-pressure range (in pascals) but also in the high-pressure range (in megapascals), as compared to other state-of-the-art sensors (*20*–*22*, *25*, *26*, *33*–*37*). To provide a more comprehensive comparison, table S2 summarizes the performance metrics of various capacitive pressure sensors, including sensitivity, hysteresis, and creep. To measure capacitive sensors and use them in various applications, we require a capacitance-to-digital converter (CDC). Using a simple electric circuit, the capacitance value can be converted into a voltage level, as illustrated in fig. S6. As shown in Fig. 4C, the relationship between *P* and the relative change of the voltage (Δ*V*/*V*_0_) for 1-MPa T3DE–based sensor shows high linearity (*R*^2^ > 0.999), which simplifies the sensor calibration process and reduces uncertainty. Our T3DE-based tactile sensor is calibrated within an analog circuit, providing a high-accuracy signal, unlike soft material–based tactile sensors that typically use digital calibration. The stable sensing performance of the T3DE-based tactile sensor is displayed in Fig. 4 (D to G). Figure 4D illustrates the reliability of our sensor, which was evaluated by applying it to 5000 repeated compression/release cycles at a pressure of 1 MPa (full-scale operation) to investigate its long-term stability and mechanical durability. No drift in the sensor response or structural changes was observed during the 5000 compression/release cycles. Specifically, the absolute percent deviations of the peaks’ average magnitudes were lower than 0.1% after 5000 cycles. To further assess the long-term durability of the sensor, we conducted an extended repeatability test up to 50,000 cycles using 1-MPa T3DE (fig. S7). The rationale behind selecting this modulus is that 1-MPa T3DE experiences higher stress concentrations at the supporting legs, making it the most rigorous test condition for mechanical stability. The results, as illustrated in fig. S1, confirm that the sensor maintains reliable performance with an absolute deviation of less than 0.1% over prolonged cyclic loading. This indicates that lower modulus variants would also exhibit comparable stability due to reduced mechanical stress during operation. Another crucial factor for the long-term stability is creep, which represents a gradual increase in deformation or strain under a constant load over time. In the case of tactile sensors, creep can cause the output signal to drift and become less accurate over time, which is a problem for long-term sensing applications. Normally, creep behavior of soft material is known to be more pronounced than that of rigid material due to the higher tan δ (loss modulus/storage modulus) of soft materials. Therefore, the T3DE-based sensor composed of rigid material exhibits less creep behavior compared to soft material–based sensors. To assess creep behavior, we conducted a 20-min test, aligning with standard practices commonly observed in commercial load cells, where short-term creep testing of over 20 to 30 min is often used as an industry benchmark for stability. This approach provides a reasonable basis for evaluating the initial stability of the T3DE-based sensor (Fig. 4E). To provide a fair comparison, we used microstructured PDMS with a Young’s modulus of ~1 MPa, which is similar to the Young’s modulus of our 1-MPa T3DE sensor. These results confirm that the sensor is suitable for repeated pressure measurement, such as long-term wrist pulse monitoring in real time. In addition to its superior resistance to creep, our T3DE-based sensor also demonstrated excellent recovery performance, as shown in Fig. 4F. After applying a high pressure of 10 MPa for 30 min, the sensor was able to recover its output signal to its original state within only 0.02 s, which is much faster than the soft elastomer–based sensor. To ensure a fair comparison, we used microstructured Ecoflex 00-30 with a Young’s modulus of ~10 kPa, which is similar to that of our 10-kPa T3DE sensor. This rapid recovery is particularly important for dynamic sensing applications, where the sensor needs to be able to respond quickly to changes in pressure. Shear resistance is another critical factor in real-world applications, particularly in wearable and interactive sensing environments. To evaluate the sensor’s robustness against lateral forces, we conducted additional shear stress tests by applying controlled lateral loads along the *x* and *y* axes. The results, presented in fig. S8, demonstrate that the sensor maintains stable performance under shear forces up to its modulus limit and fully recovers after stress removal. Given that we used a thermoplastic film as the substrate for the T3DE, the temperature sensitivity of the T3DE-based tactile sensor is inevitable. However, the biaxially oriented PET (BoPET) substrate that we used for our platform has high temperature stability (fig. S9), enabling the sensor to maintain its performance up to 60°C (Fig. 3G). This is particularly noteworthy, as most electronic components used in tactile sensors are not designed to withstand temperatures exceeding 60°C. Moreover, our sensor demonstrated consistent performance across various humidity conditions. To quantify the impact of environmental factors, we conducted additional durability tests under extreme humidity and temperature conditions (fig. S10). We examined the limit of detection (LOD) of tactile sensor based on T3DE, as shown in Fig. 4H. The LOD is an important parameter for the realization of ultralow force detection capabilities, such as monitoring of air flows, sound waves, and propagation of small mechanical vibrations. We estimated the LOD to demonstrate the ultralow pressure detection capability of the 30-Pa T3DE sensor, suitable for fine airflow and acoustic wave applications. For the test, we placed an ultralightweight thin film (~0.01 mg, equivalent to ~0.02 Pa) on the sensor, and it was able to detect the film without notable noise. Using the baseline noise level and a signal-to-noise ratio of 3, we estimated the LOD to be ~0.0015 Pa. For the characterization of dynamic responses to ultralow pressure, we applied 10-kHz acoustic wave (~60 dB) to the sensor (Fig. 4I). Here, the response time is defined as the duration it takes for the output signal to change. The 30-Pa T3DE–based sensor has a much shorter response time, enabling it to accurately detect a 10-kHz acoustic wave, which corresponds to a wave period of ~0.1 ms.

### Application of T3DE-based tactile sensors to detect a wide spectrum of pressure levels

**Fig. 5.**
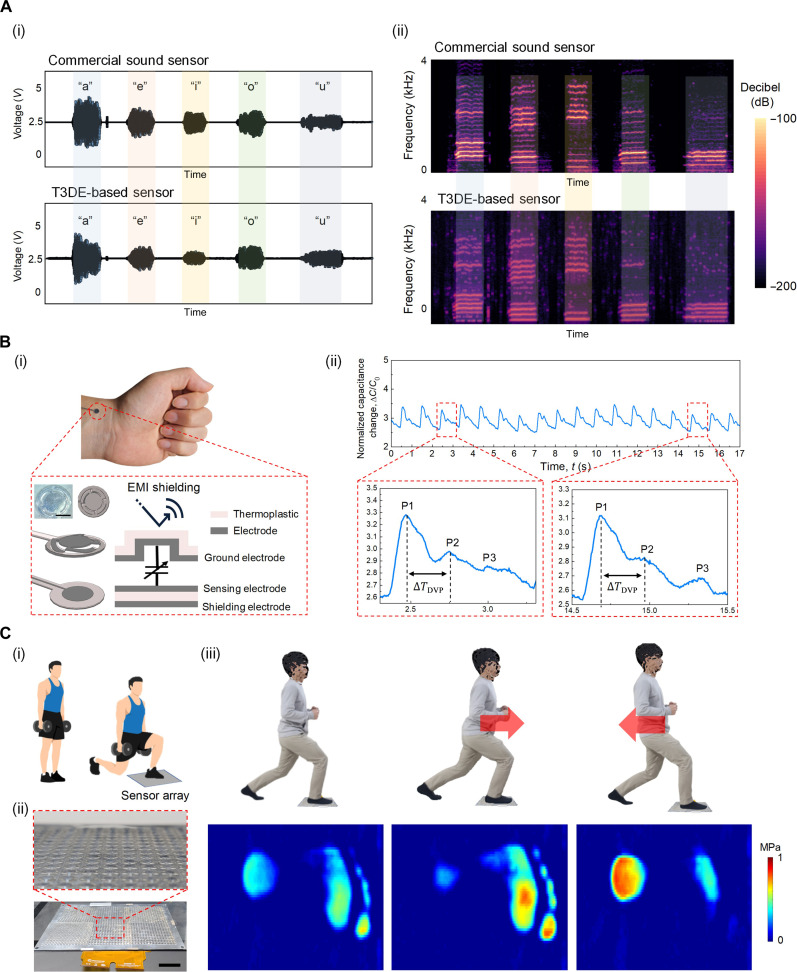
Versatile applications of T3DE-based tactile sensors. (**A**) Demonstration of sound sensor based on the 30-Pa T3DE–based tactile sensor. (i) Acoustic waveforms measured by the commercial sound sensor and the 30-Pa T3DE–based tactile sensor. Output signals of these sensors have similar tendency when speaking different words. (ii) Spectrograms of the commercial sound sensor and the 30-Pa T3DE–based tactile sensor. Similar characteristic peaks and patterns of the spectrograms can be found between the two sensors. (**B**) Demonstration of blood pulse sensor based on the 5-kPa T3DE. (i) Sensor design for detecting blood pulse waves on the wrist with the implementation of shielding. Scale bar, 2 mm. (ii) Real-time blood pulse monitoring on the wrist. The designed sensor clearly detected the blood pulse waves on the wrist with high sensitivity, which can obtain distinguishable peaks (P1, P2, and P3). (**C**) Demonstration of workout monitoring systems based on the 1-MPa T3DE. (i) Schematic illustration of workout monitoring systems. The sensor array can measure a plantar pressure distribution, which can be useful to improve posture and understand target muscular activation during the workout. (ii) Optical images of high-resolution sensor array with 40 pixels by 70 pixels (each pixel has 6 mm by 6 mm of spatial resolution). Scale bar, 5 cm. (iii) Plantar pressure distributions during lunge exercise. Photo credit: J. Choi, Electronics and Telecommunications Research Institute (ETRI).

To demonstrate the utility of our method for detecting a wide spectrum of pressure levels, we developed and implemented custom-designed T3DE-based sensors in various tactile sensing applications, including the sound sensor (approximately pascals), the blood pulse sensor (approximately kilopascals), and the workout monitoring system (approximately megapascals). In the case of the sound sensor, we used a 30-Pa T3DE–based tactile sensor capable of detecting ultralow pressure range with a fast response. Figure 5A presents a comparison between the acoustic waveforms obtained from our sensor and the commercial sound sensor. Both sensors exhibited similar tendencies when different words such as “a,” “e,” “i,” “o,” and “u” were spoken at an approximate sound level of 70 dB (Fig. 5A-i). To further analyze the captured sound waves, we converted the output signals into spectrograms, which provide a visual representation of the frequencies over time. Notably, Fig. 5A-ii shows spectrograms with similar characteristic peaks and patterns. These results highlight the potential of our sensor in applications such as voice recognition and speech visualization, which can benefit individuals with hearing impairments. In addition to the sound sensor application, another crucial area where our method proves its utility is in blood pulse sensing for wearable health care applications. Typically, the pressure range for blood pulse sensing is around 1 kPa (*21*, *25*). In our study, we designed and fabricated a sensor based on a 5-kPa T3DE, which exhibits high sensitivity in the vicinity of 1 kPa. Figure 5B illustrates the sensor design for detecting blood pulse waves on the wrist. To address electromagnetic interference (EMI) caused by the human body, we implemented an active shielding method, covering the sensing electrode with a ground and shielding electrode (Fig. 5B-i). Consequently, the designed sensor accurately detected blood pulse waves on the wrist with high sensitivity (Fig. 5B-ii). The measured blood pulse wave yielded three distinguishable peaks (P1, P2, and P3). Using these peaks, the radial artery augmentation index (AIr = P2/P1) and the transit time (Δ*T*_DVP_), defined as the time difference between P1 and P2, can be calculated. These parameters can be used to estimate clinical metrics such as blood pressure, cardiac output, arterial stiffness, vascular age, and overall heart-health indicators (*38*). In addition, when compared to commercial sensors, our design demonstrates superior performance in detecting blood pulse waves owing to its high sensitivity and ability to accurately capture critical waveforms. This makes it advantageous for precise measurements and clinical applications (fig. S11). In expanding the application of our method to high-pressure scenarios, particularly those involving exercises such as lunges, we effectively demonstrated the use of a pressure sensor array to monitor plantar pressure distribution, as shown in Fig. 5C. According to the literature, plantar pressure during these activities can range from ~100 to 500 kPa and can even exceed 700 kPa in certain regions of the foot during high-intensity activities (*39*). Understanding this distribution is critical for improving posture and gaining insights into targeted muscle activation during exercise. The sensor array was engineered to perform exceptionally well in high-pressure environments and can be manufactured on a large scale. To map the plantar pressure distribution, we constructed a high-resolution sensor array with a spatial resolution of 6 mm by 6 mm per pixel, encompassing a total of 40 pixels by 70 pixels. This sensor array also includes EMI shielding to minimize EMI, ensuring accurate pressure measurements. We also implemented an interpolation technique to visualize the pressure distribution and estimate unknown pressure levels in adjacent cells based on a limited dataset. This method enhanced the resolution to 1 mm by 1 mm per pixel, covering a grid of 240 pixels by 280 pixels. The high sensitivity and resolution of our sensor array, combined with the interpolation technique, allow for the detection of minute variations in plantar pressure during lunge exercises, as demonstrated in Fig. 5C-ii). By addressing challenges such as EMI and ensuring high performance, our approach offers a viable and effective solution for commercialization and widespread use in various application fields such as sports, medicine, and wearable electronics.

### Demonstration of T3DE-based tactile sensor array for augmented reality system

**Fig. 6.**
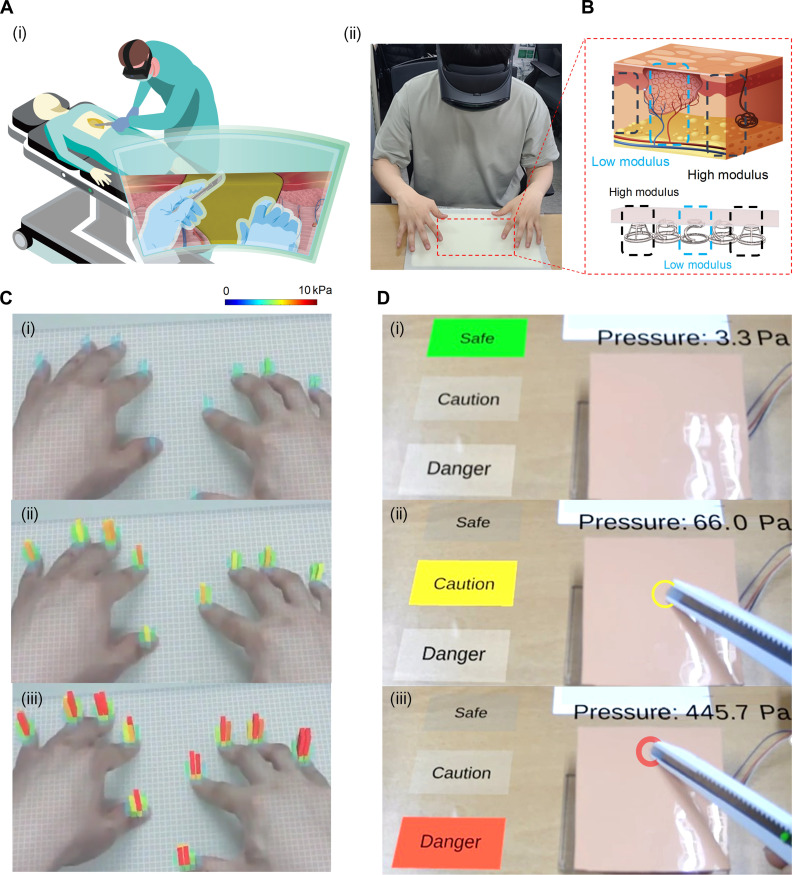
Integration of AR with T3DE-based tactile sensors. (**A**) Schematic (i) and optical (ii) images demonstrate the AR system for potential applications such as surgical training. (**B**) Customization of T3DE-based sensor array to mimic real tissue models. Our fabrication method can achieve a specific Young’s modulus of each component in the artificial tissue model. (**C**) Sequential images of the tactile interaction with the sensor array as a response to applied pressures (i to iii) within the AR environment. (**D**) Visual and tactile feedback from the T3DE sensor array at varying pressure levels. The center of the array uses a more flexible sensor to represent softer tissue, while the outer edge uses a stiffer sensor to simulate harder tissue. The sensor array provides visual feedback at three pressure thresholds—“Safe,” “Caution,” and “Danger” (i to iii)—to guide users on appropriate force application during procedures. Photo credit: J. Choi, Electronics and Telecommunications Research Institute (ETRI).

We present potential applications of our sensor array configuration to augmented reality (AR), such as a surgery stability training system (Fig. 6A). Traditional surgical training typically relies on expensive in-person laboratories using cadaver dissections, which are costly and have become even more challenging because of heightened ethical standards and restrictions in today’s world. Unlike traditional methods, AR enhances the training experience by augmenting and overlaying the real clinical environment, thereby enriching the surgeon’s experience without isolating them in a virtual world. Consequently, AR systems for surgery stability training have been developed, primarily providing visual and auditory feedback. However, these training systems would benefit from incorporating force and motion feedback to simulate stiffness and other tactile sensations for more immersive and natural interactions. Our fabrication method allows for the customization of Young’s modulus for each tactile sensor in the sensor array. This capability enables the fabricated sensor array to mimic the real tissue structure beneath human skin. Each sensor component in the array provides mechanical feedback and pressure levels, assisting the surgeon in analyzing surgical stability. Figure 6B illustrates real tissue systems, noting that tissue stiffness varies because of different diseases. This variability can be modeled as a mechanical system using springs with different stiffness levels. Initially, to demonstrate a visual feedback test, we used a 40-by-70 sensor array. This array was created using an interpolation method, as depicted in Fig. 6C. The figure sequentially displays images of tactile interactions within the AR environment, capturing responses to applied pressures (movie S1). Next, in applying this technology for a surgery stability training system, the middle area of the tactile sensor was designed to mimic soft tissue (with a Young’s modulus of approximately kilopascals), and the outer sensor was designed to mimic hard tissue (with a Young’s modulus of approximately megapascals), as shown in Fig. 6D. Furthermore, this system incorporates a stiffer sensor structure at the outer edge of the array to mimic the feel of hard tissue, allowing users to experience the distinct stiffness as a tactile feedback alongside a visual feedback. This combination enables the system to provide both visual and tactile feedbacks in real time, enhancing excision stability training by informing users on the varying stiffness of tissues and guiding their actions based on the sensor feedback during operations. An additional visual demonstration illustrating how modulus variation translates to tactile perception is shown in fig. S12. For demonstration, we simulated a human skin–like cover on the sensor array. Sensor outputs increase continuously with applied pressure during excisions, and the variation in sensor signals can be used to indicate the stability of cutting events. For various purposes, the pressure level threshold can be adjusted. In this case, we set a range for stable pressure and a range for dangerous pressure levels that are too high for safe cutting (movie S2). The proposed system provides an AR platform that allows surgical trainees to simulate and train on the excision of various tissues or tumors with different stiffness levels.

## DISCUSSION

This paper introduces a design and manufacturing method for tactile sensors, using modulus-tunable T3DE that provides high sensing performance and customizability. Our approach begins with laser-based electrode patterning and precision structuring of thermoplastic films, followed by a thermoforming process that transforms these 2D patterned films into 3D structures with tunable mechanical properties, particularly modulus. This tunability allows us to adjust the sensor’s stiffness based on application needs, enhancing its functionality and adaptability. By combining laser patterning with thermoforming, our method simplifies the fabrication process, making it more cost-effective, scalable, and suitable for mass production compared to traditional approaches. Furthermore, T3DE provides superior creep stability and recovery speed as compared to soft elastomer–based devices. We demonstrated T3DE-based tactile sensors in diverse applications, including sound and pulse monitoring, workout tracking, and AR systems, underscoring the versatility and practical value of this technology.

However, there are still areas that need further exploration and improvement. First, optimizing the thermoplastic materials and sensing electrodes could enhance sensor performance and durability. Second, developing high-resolution patterning and thermoforming equipment will improve the precision and scalability of the manufacturing process. In addition, comprehensive studies on the various parameters of T3DE, such as different geometries and material properties, are necessary to fully understand and exploit the capabilities of these sensors. Furthermore, thermoplastic materials are sensitive to heat, which limits their application in high-temperature environments. The 3D structured configuration may also exhibit different behaviors under shear forces, requiring further investigation to ensure reliability under various loading conditions.

Research into 3D structuring of rigid film materials is advancing rapidly owing to their potential for high precision, durability, and versatile applications. These developments enable processes and studies with various material properties, which can be beneficial in applications such as aerospace, automotive, and wearable technology. Inspired by these advances, our work aims to contribute to the broader field of tactile sensor technology and affect diverse applications beyond traditional tactile sensing, including robotics, health care, and interactive systems (fig. S13). Furthermore, while vision-based and machine learning applications have advanced, tactile sensors have lagged because of performance and validation challenges. Our research aims to bridge this gap, enabling more effective integration of tactile sensors in these fields. Future research should focus on these aspects to advance the field. Investigating long-term stability and environmental effects, such as temperature and humidity variations, will be crucial for real-world applications. Exploring integration with machine learning algorithms for data interpretation and enhancement of sensor functionality can further extend the utility of these tactile sensors. By addressing these challenges, we can move toward a quick, high-performance, low-cost, and scalable manufacturing process for tactile sensors, substantially contributing to the development of tactile sensor technology with more advanced functionalities.

## MATERIALS AND METHODS

### Materials

The primary materials used in the fabrication of T3DE included high-performance thermoplastic films, such as BoPET, which was purchased from SheetMeka (South Korea). However, other types of thermoplastic films can also be effectively used for this purpose. For bonding the films, we used a thermal bonding film (3M 583) from 3M (USA). Most commercially available urethane- and epoxy-based adhesives work well in this process. Similarly, a variety of commercially available thermal bonding films and anisotropic conductive films, such as Anisotropic Conductive Film (ACF) 7303 from 3M (USA), are compatible with these applications. The thermoforming mold was made from aluminum, selected because of its excellent thermal conductivity and machinability. The mold is designed to withstand high temperatures, specifically above 250°C, ensuring its suitability for high-temperature thermoforming processes. This thermal resistance ensures the mold’s durability and stability even under prolonged exposure to elevated temperatures during manufacturing.

### T3DE-based tactile sensor fabrication process

The fabrication process of the T3DE-based tactile sensor (fig. S14) began with laser patterning of the electrodes and thermoplastic film. In this step, the electrode design and film structure were precisely defined on the surface of the thermoplastic material, forming the top and bottom substrates. We achieved this by adjusting the laser power to selectively pattern both the electrode and the plastic film (fig. S15). The patterned films were then subjected to a thermoforming process, in which they were placed into aluminum molds and underwent elastic deformation under a controlled pressure of 10 MPa and a temperature of 180°C for a duration of 5 min. This was followed by stress relaxation through controlled heating, enabling the formation of the 3D structure required for the sensor. The molds were meticulously prepared with positive and negative shapes made from aluminum or other suitable metals, and the films were aligned using predefined alignment marks to ensure accuracy during assembly (fig. S16). Once thermoforming was completed, the 3D structure was attached to a bottom plate using a thermal bonding process (fig. S17). During this step, the temperature was carefully controlled to remain at 60°C, preventing damage to the materials while ensuring strong adhesion between the T3DE structure and the bottom plate. Various adhesives were selected on the basis of material compatibility for this bonding process to complete the assembly of the capacitive tactile sensor array that uses air as the dielectric. As an example of this process, a single-channel miniature pressure sensor was fabricated by laser patterning the T3DE layer, adhesive layer, and bottom plate. These components were aligned using alignment marks, pressed together, and laser cut to achieve the desired sensor shape. Last, connectors were attached to complete the assembly, resulting in a functional capacitive sensor (fig. S18).

### Finite element analysis

FEA was conducted using ABAQUS software (Dassault Systèmes Simulia Corporation, USA) to evaluate the mechanical behavior of the T3DE structure fabricated through thermoforming (fig. S19). Initially, the simulation assumed that the thermoplastic film would undergo elastic deformation during the thermoforming process. Once the deformation was achieved, it was further assumed that the material would retain its shape upon heating and subsequent cooling. This assumption was crucial to ensure that the electrodes would not be damaged during the thermoforming process. The structure was modeled with a circular geometry to match the actual design of the sensors. In the second phase of the simulation study, various design parameters, such as the number and length of the supporting legs, were explored to optimize the sensor’s mechanical properties. These parametric studies helped determine how variations in leg geometry influenced key mechanical properties, such as stress distribution and Young’s modulus, which are critical for sensor sensitivity.

### Characterization of T3DE-based tactile sensor

The characterization of the tactile sensors involved mechanical tests to measure the modulus of elasticity, hysteresis, and creep under cyclic loading conditions. A high-precision universal testing machine (AGS-X, SHIMADZU, Japan) was used to evaluate the sensing performances. Disk-type compression fixtures with diameters of 40, 20, 10, and 5 mm were used for uniform deformation of the sensor. The force was recorded with a force transducer, ensuring ±0.5% accuracy of the readout value, with a maximum load capacity of 1 kN. The capacitance of each sensor was measured using a precision inductance–capacitance–resistance (LCR) meter (E4980A, Agilent, USA) at a frequency of 400 kHz. A commercial CDC (PCap04, ScioSense B.V., Netherlands) was used to record and digitize the capacitance of the sensor. To convert the capacitance to voltage, a high-precision operational amplifier (LMP7721, Texas Instruments, USA) was used, enabling accurate signal amplification. The microcontroller unit (MCU) controlled a switch to alternately charge and discharge the sensor, while the resulting voltage was measured using an external 24-bit ADC. This setup allowed for precise capacitance-to-voltage conversion, which was then calibrated to pressure using a predetermined calibration curve. The capacitance and voltage relationship exhibited a linear response, resulting in a linear pressure-voltage characteristic, which simplifies signal interpretation and sensor calibration.

### Demonstration of T3DE-based tactile sensor for various applications.

The sound sensor demonstration incorporated a T3DE sensor with an ultralow modulus to detect subtle pressure changes induced by acoustic waves. A capacitance-to-voltage conversion circuit was used to process the sensor’s output and enhance responsiveness. The output from the T3DE sensor was captured and analyzed using a digital acquisition system for precise data collection. Spectral data from the sensor were visualized and analyzed with spectral analysis software from Ocean Optics (USA), supporting its use in applications requiring sensitive detection, such as spectrometry. For the pulse measurement, a single-channel sensor was fabricated as described in fig. S5. A commercial SingleTact capacitive pressure sensor (Pressure Profile Systems, USA) was used as a benchmark to validate the performance of the T3DE-based pulse sensor, confirming its accuracy in detecting subtle changes in pulse pressure. In the AR system, a T3DE sensor array was configured to simulate haptic feedback for surgical training. Instead of using a physical haptic actuator, we adjusted the modulus of each T3DE sensor in the array to mimic different tissue stiffness levels, allowing users to experience a tactile response that corresponds to various tissue properties, such as softness and elasticity. The system was integrated with Unity (Unity Technologies, USA) to create an immersive 3D environment, enabling users to simulate surgical procedures and interact with virtual tissues of varying stiffness. This setup provided a realistic training platform, allowing users to feel simulated tactile feedback directly influenced by the mechanical properties of the T3DE sensors themselves, without the need for an external haptic device. The experiment was approved by the institutional review board of Korea Advanced Institute of Science and Technology (IRB no. KH2021-116).
