# Supplementary material for: Thermoforming 2D films into 3D electronics for high-performance, customizable tactile sensing
Source: Sci Adv. 2025 May 14;11(20):eadv0057. doi: 10.1126/sciadv.adv0057 (PMC12077495; doi:10.1126/sciadv.adv0057)
Supplement: Supplementary file 1 — Figs. S1 to S19 Tables S1 and S2 Legends for movies S1 and S2 [file sciadv.adv0057_sm.pdf]

Science Advances
AAAS

Supplementary Materials for

Thermoforming 2D films into 3D electronics for high-performance, customizable tactile sensing

Jungrak Choi *et al.*

Corresponding author: Inkyu Park, inkyu@kaist.ac.kr

Sci. Adv. **11**, eadv0057 (2025)
DOI: 10.1126/sciadv.adv0057

The PDF file includes:

Figs. S1 to S19
Tables S1 and S2
Legends for movies S1 and S2

Other Supplementary Material for this manuscript includes the following:

Movies S1 and S2

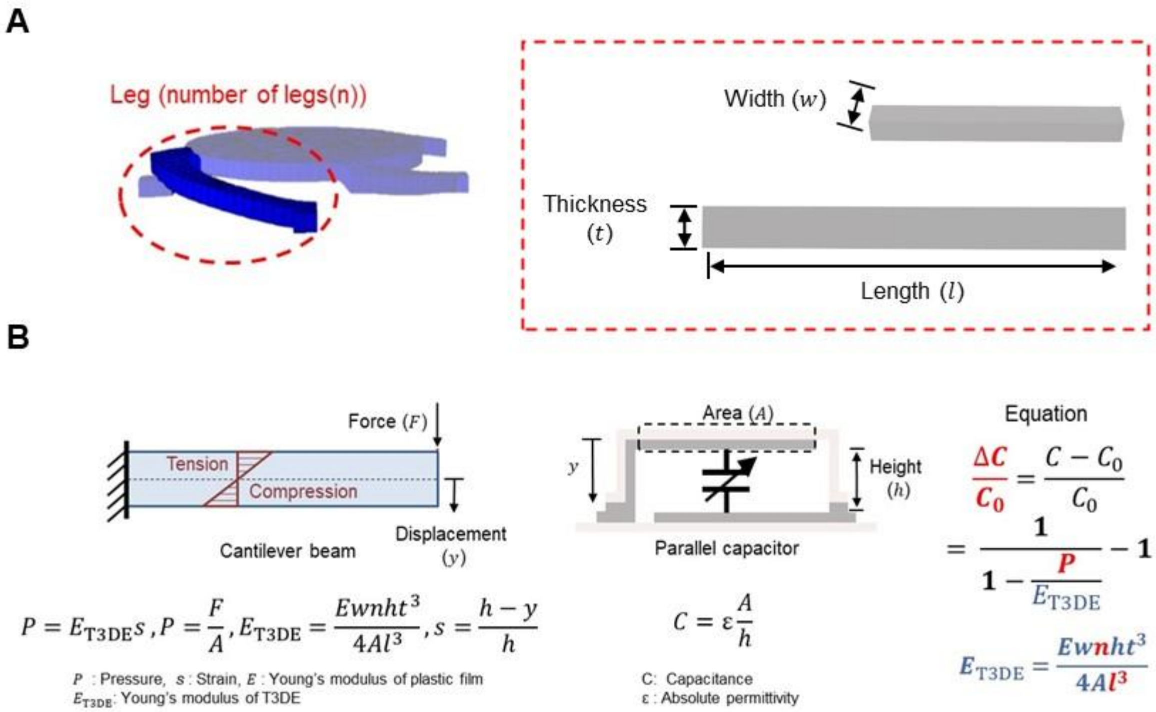

Fig. S1. Analytic solution for the T3DE-based sensor. (**A**) The legs of the T3DE structure are modeled using beam bending theory, assuming small deformation. (**B**) The analytic solution uses the beam bending formula combined with the parallel plate capacitor equation, allowing for the calculation of the effective Young's modulus for each T3DE structure.

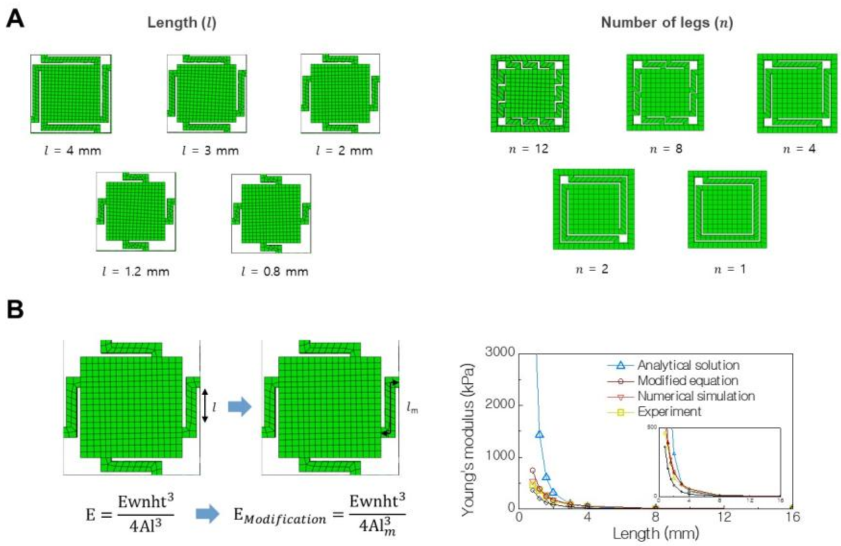

Fig. S2. Structural Optimization Using Finite Element Analysis (FEA). (**A**) FEA modeling of simplified rectangular structures to analyze the effect of leg length and number of legs on mechanical response. (**B**) Comparison of theoretical models, numerical simulations, and experimental results, incorporating length correction to improve agreement between theoretical predictions and actual measurements.

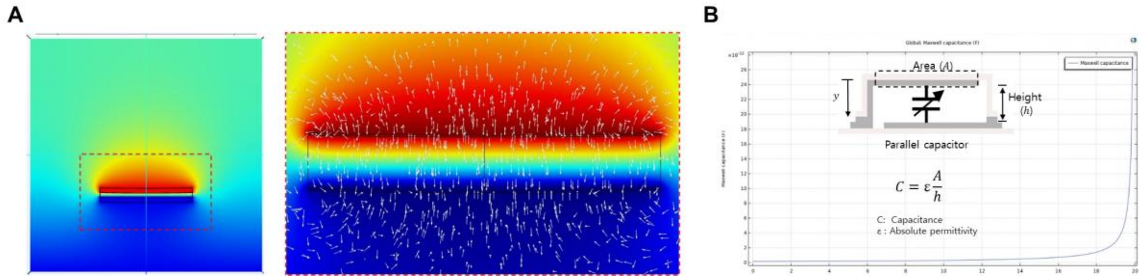

Fig. S3. Electrostatic simulation and capacitance modeling of the T3DE sensor. (**A**) COMSOL-based electric field distribution and potential gradient analysis under applied pressure. (**B**) Theoretical capacitance modeling using the parallel plate capacitor equation, confirming the agreement between simulation results and analytical predictions.

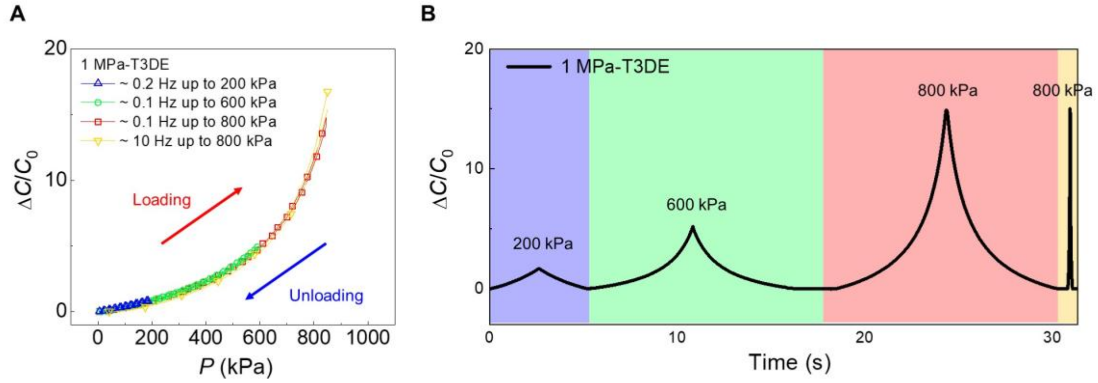

Fig. S4. Cyclic capacitance response of the 1 MPa-T3DE sensor under different force and frequency conditions. (**A**) Capacitance variations were measured under cyclic loading at 0.1 Hz with maximum pressures of 200 kPa, 600 kPa, and 800 kPa, as well as at 10 Hz up to 800 kPa. The 10 Hz test was selected as it represents the fastest achievable cyclic loading using conventional force measurement equipment. For higher-speed force measurements, acoustic-based methods must be utilized. The results confirm that the sensor maintains a stable response with minimal hysteresis, demonstrating its suitability for dynamic force sensing applications. (**B**) Time-dependent capacitance response of the sensor under cyclic loading.

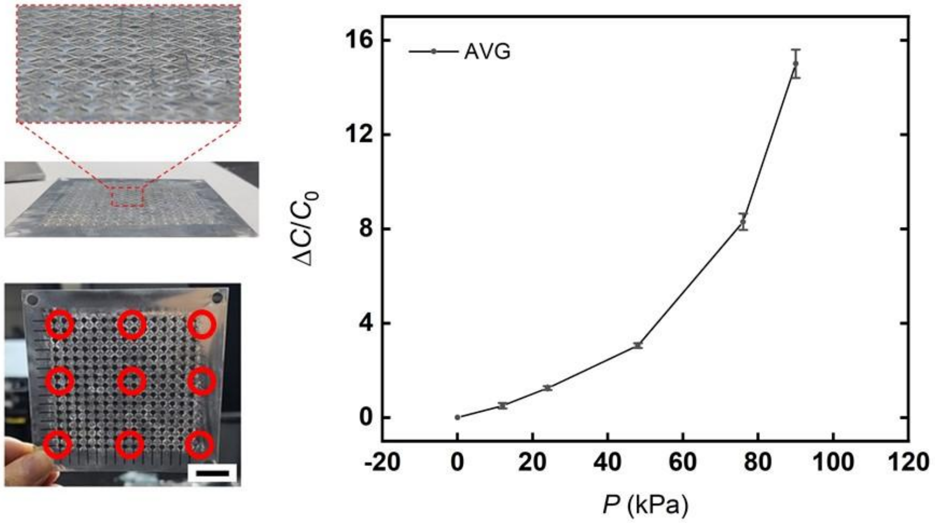

Fig. S5. Statistical analysis of capacitance responses across the T3DE sensor array. Measurements were taken from 9 positions, including the center, edge, and intermediate regions, under a pressure of 0-100 kPa. The results confirm high uniformity, with performance variations within 5% across different sensor positions. Scale bar: 20 mm. Photo Credit: Jungrak Choi, ETRI.

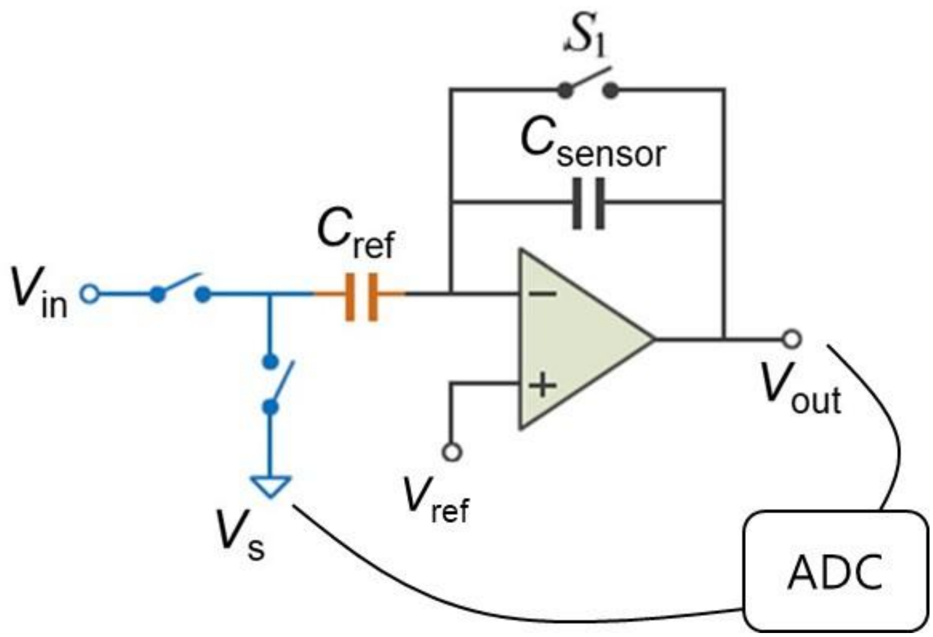

Fig. S6. Capacitance measurement circuit. This circuit uses a switched-capacitor configuration to convert changes in the sensor's capacitance (C_{sensor}) to a voltage output (V_{out}). By setting the ADC reference voltage to match the sensor's initial output, the system can start readings at zero, optimizing the full ADC range for detecting capacitance changes due to applied pressure.

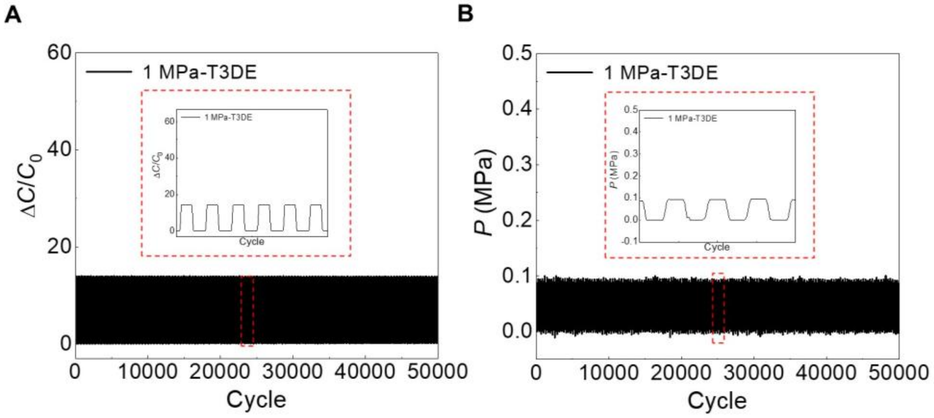

Fig. S7. 50000 cycles repeatability test results for 1MPa-T3DE. (**A**) Normalized capacitance change ($\Delta C/C_0$) over 50000 cycles, demonstrating high repeatability. (**B**) Applied pressure (P) during cyclic loading, confirming stable mechanical response.

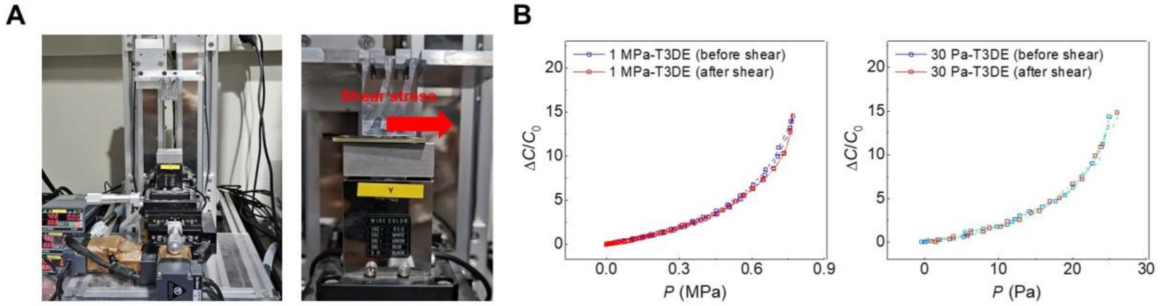

Fig. S8. Experimental setup and results of shear stress testing. (**A**) Experimental setup illustrating shear stress application along the x- and y-axes on the T3DE sensors. (**B**) Measured capacitance response of the T3DE sensor before and after shear stress application, confirming that the sensor remains stable and fully recovers after stress removal. Photo Credit: Jungrak Choi, ETRI.

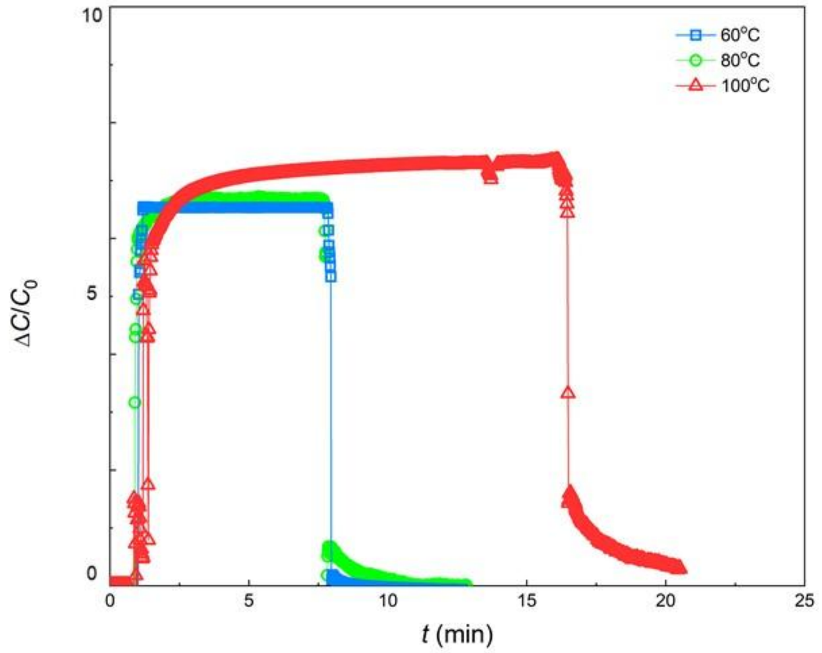

Fig. S9. Temperature stability analysis of the sensor. The sensor was subjected to constant pressure at 60°C, 80°C, and 100°C, and the corresponding signal was recorded over time. As the temperature increased, the sensor exhibited a progressive rise in signal, indicating increased compression under thermal expansion. At 60°C, negligible signal variation was observed, demonstrating excellent stability. Even at 80°C, the sensor maintained reliable performance with minimal drift, indicating its robustness under moderate thermal conditions.

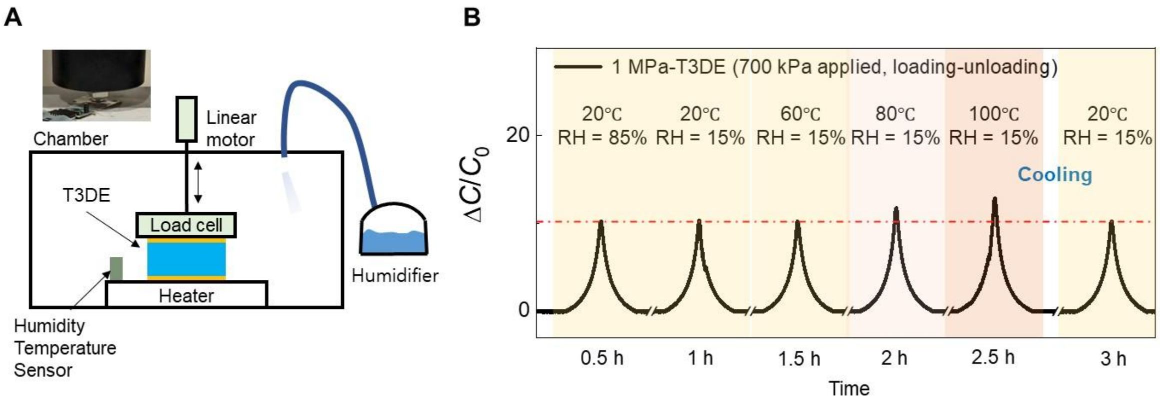

Fig. S10. Environmental stability test results for 1 MPa-T3DE sensor. The sensor was tested under varying temperature and humidity conditions to evaluate its robustness against environmental fluctuations. (**A**) Schematic representation of the experimental setup. (**B**) Capacitance response of the sensor under different relative humidity (RH) and temperature conditions. The sensor's response was recorded every 30 minutes after each temperature change to ensure thermal and humidity stabilization before measurement. The sensor was first exposed to 85% RH at 20 °C, followed by a reduction to 15% RH. Temperature variations from 20 °C to 100 °C were applied while maintaining 15% RH, and the sensor's response was recorded. The cooling process was also monitored to assess performance recovery. Photo Credit: Jungrak Choi, ETRI.

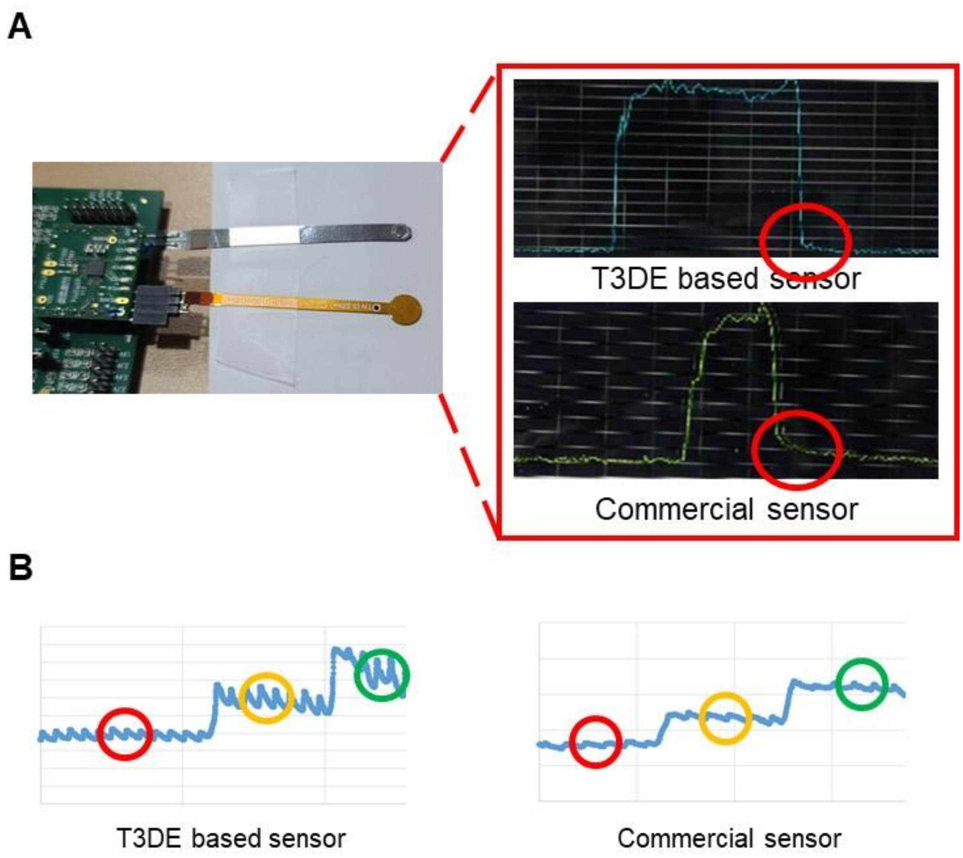

Fig. S11. Performance comparison between the T3DE sensor and a commercial sensor (SingleTac). (**A**) Recovery response after pressure application and release, showing that the T3DE sensor exhibits faster recovery compared to the commercial sensor. (**B**) Pulse wave measurement, where the T3DE sensor demonstrates clearer signal detection as pressure increases, while the commercial sensor struggles to capture the signal accurately. This highlights a significant advantage of the T3DE sensor, particularly over soft material-based sensors, which typically experience reduced sensitivity under increased pressure. In contrast, the T3DE sensor shows improved sensitivity with higher pressure, making it well-suited for such applications. Photo Credit: Jungrak Choi, ETRI.

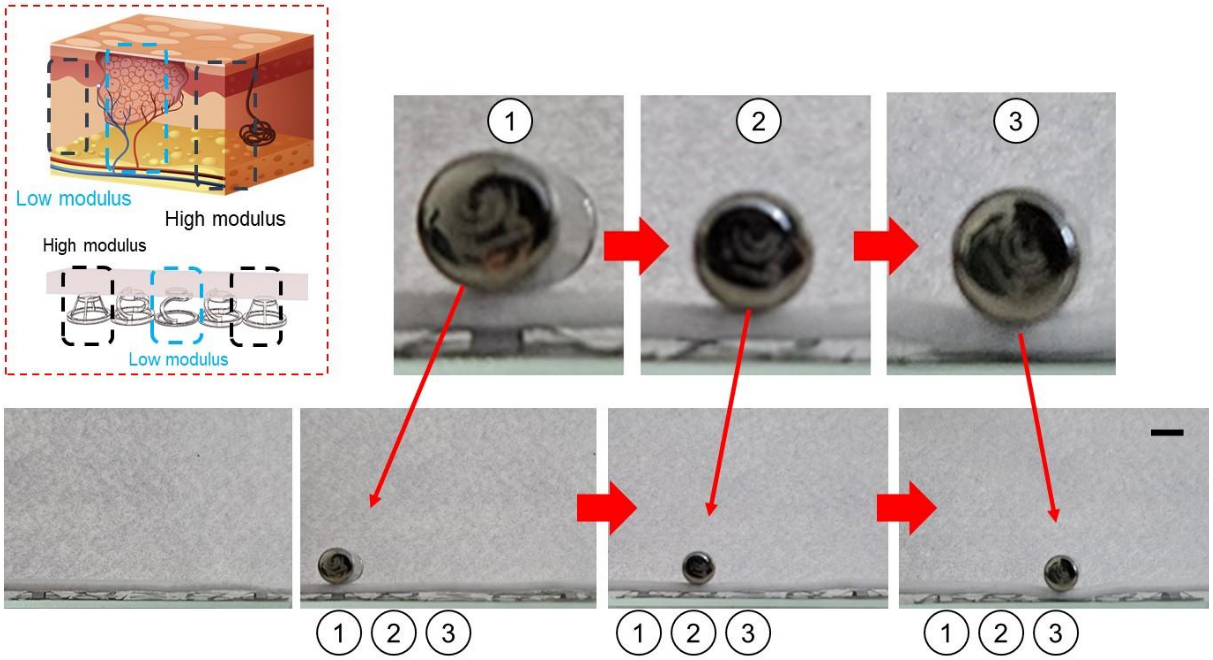

Fig. S12. Visual demonstration of T3DE sensors with different Young's moduli arranged in an array, showing varying deformation when an object is placed on top, illustrating the sensor's ability to mimic tactile sensations of different stiffness levels. (**A**) Schematic representation of T3DE sensors with different Young's moduli, mimicking biological tissues of varying stiffness. The illustration shows how high-modulus sensors resist deformation while low-modulus sensors allow greater compression under the same applied force. (**B**) Experimental demonstration of the deformation behavior of T3DE sensors under an applied load. The numbered images represent sequential deformation states as the object is placed and removed from the sensors. The results indicate that materials with lower Young's modulus exhibit greater deformation, whereas higher modulus materials retain their shape more effectively. This highlights the capability of T3DE sensors to replicate real-world tactile stiffness variations, providing a fundamental basis for haptic and augmented reality applications. Scale bar: 3 mm. Photo Credit: Jungrak Choi, ETRI.

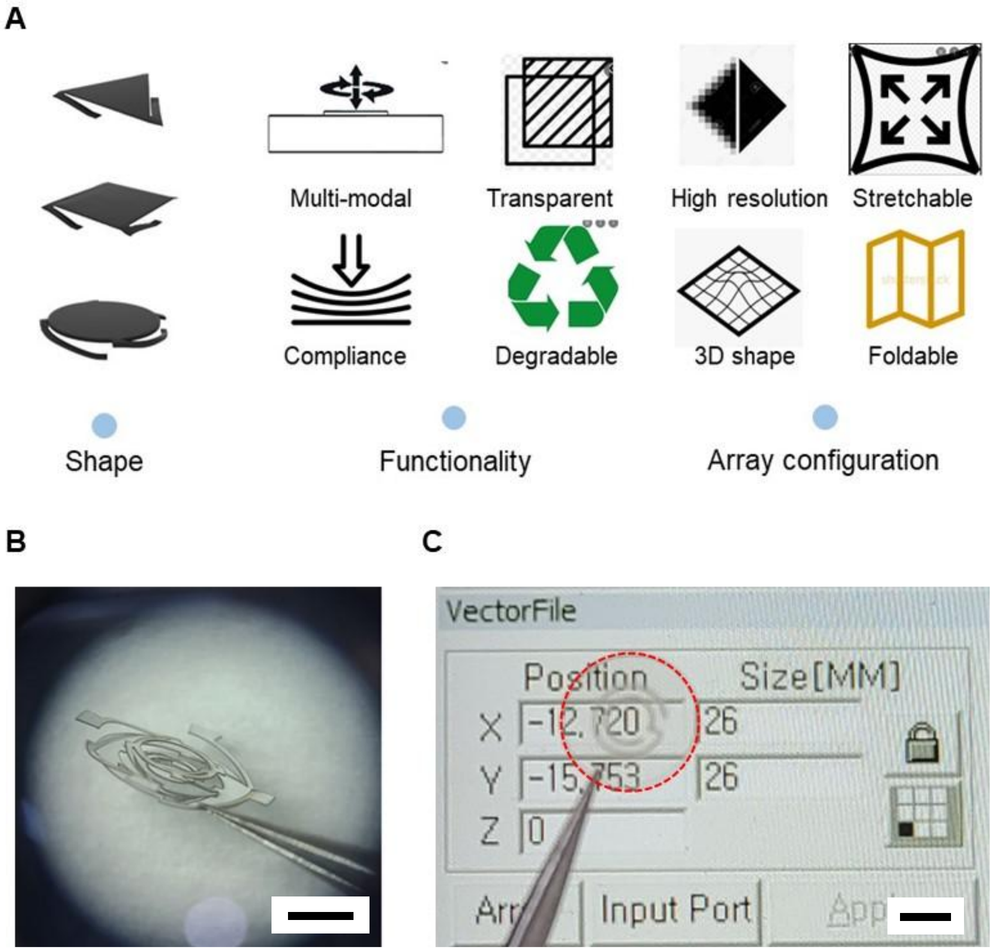

Fig. S13. Versatile potential applications of the T3DE sensor. (**A**) Shape, functionality, and array configuration flexibility, allowing the sensor to be adapted for a wide range of tactile sensing applications. (**B**) Example of a complex shape designed to function as a strain and compliance sensor, demonstrating the T3DE's adaptability to various geometries. Scale bar: 5 mm. (**C**) Use of different thermoplastic materials, such as transparent substrates, enabling the development of transparent pressure sensors for specific applications. Scale bar: 2 mm. Photo Credit: Jungrak Choi, ETRI.

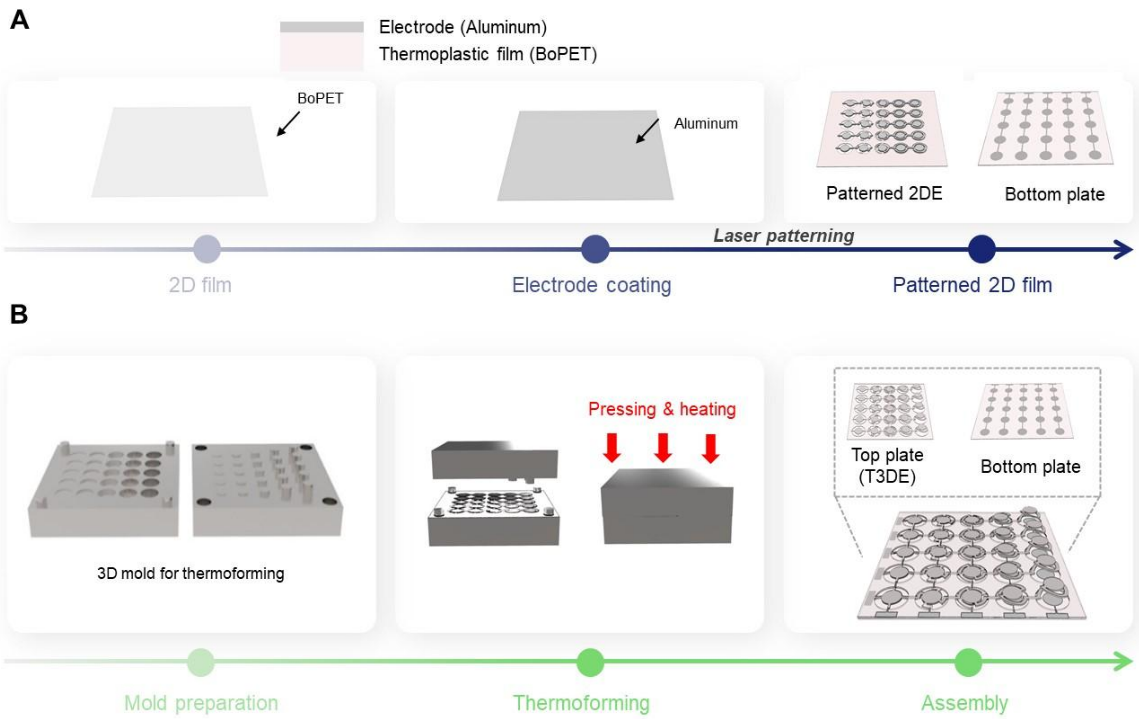

Fig. S14. Fabrication process of the T3DE-based tactile sensor. (**A**) Laser patterning of the electrode and thermoplastic film, where the desired electrode and film design is created on the surface, forming the top and bottom substrates. (**B**) Thermoforming and assembly process, in which the patterned films are placed in a mold and undergo elastic deformation through applied pressure, followed by stress relaxation via heat. The 3D structure is then attached to a bottom plate, finalizing the assembly of the T3DE-based tactile sensor array, utilizing a capacitive sensing mechanism with air as the dielectric.

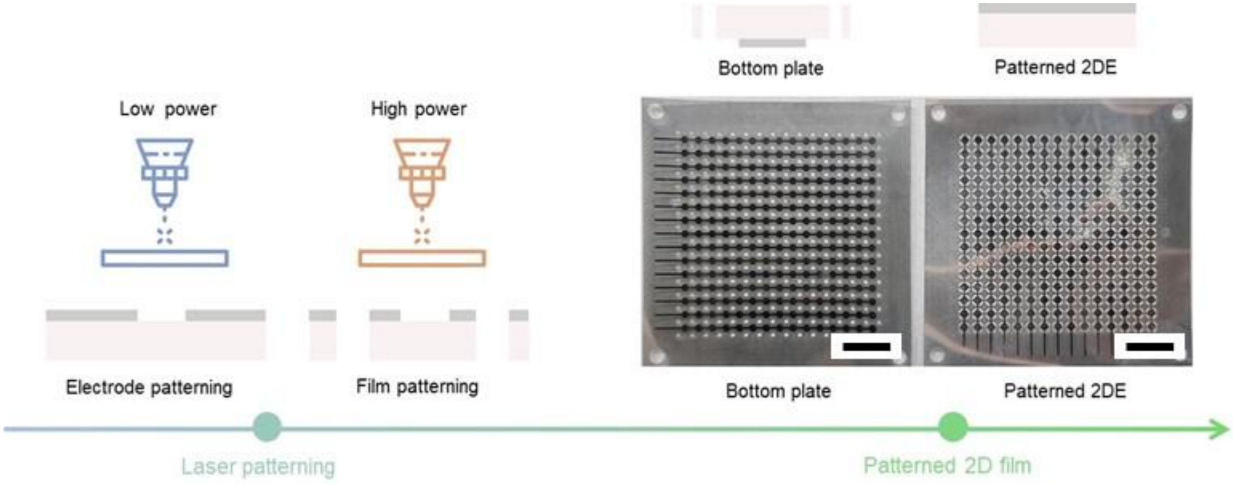

Fig. S15. Selective patterning process for the electrode and plastic film using laser power adjustments. By fine-tuning the laser's power, both the electrode and the plastic film can be selectively patterned, enabling the production of the patterned 2D electronics (2DE) for thermoforming and the bottom plate required for sensor assembly. Scale bar: 20 mm. Photo Credit: Jungrak Choi, ETRI.

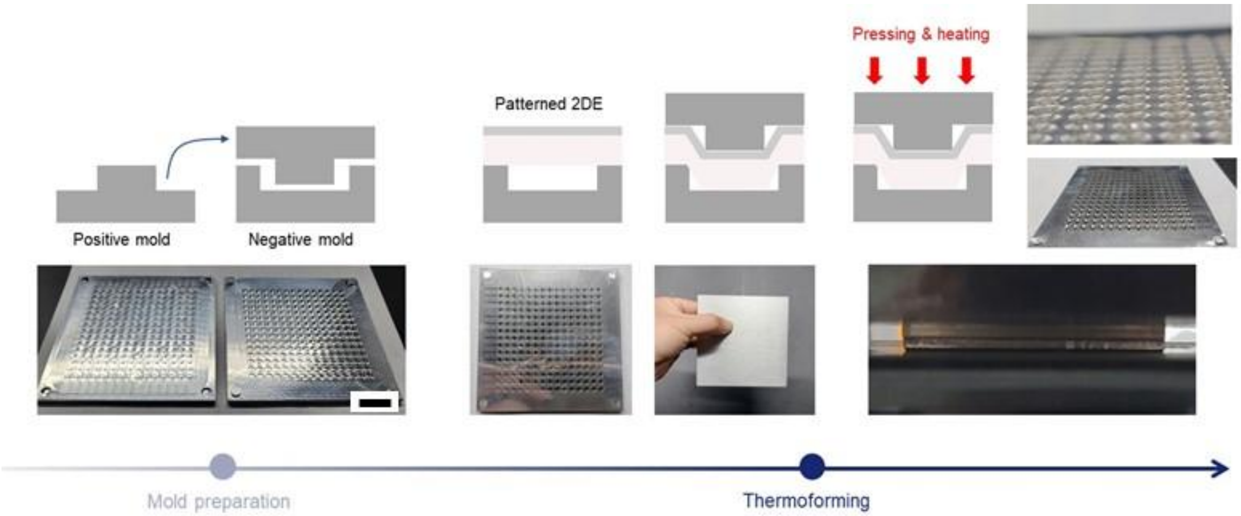

Fig. S16. Mold preparation and thermoforming process. Positive and negative molds made from aluminum or other metals are prepared. The patterned 2D electronics (2DE) are then precisely aligned using alignment marks, and pressure and heat are applied to fabricate the T3DE structure. Scale bar: 20 mm. Photo Credit: Jungrak Choi, ETRI.

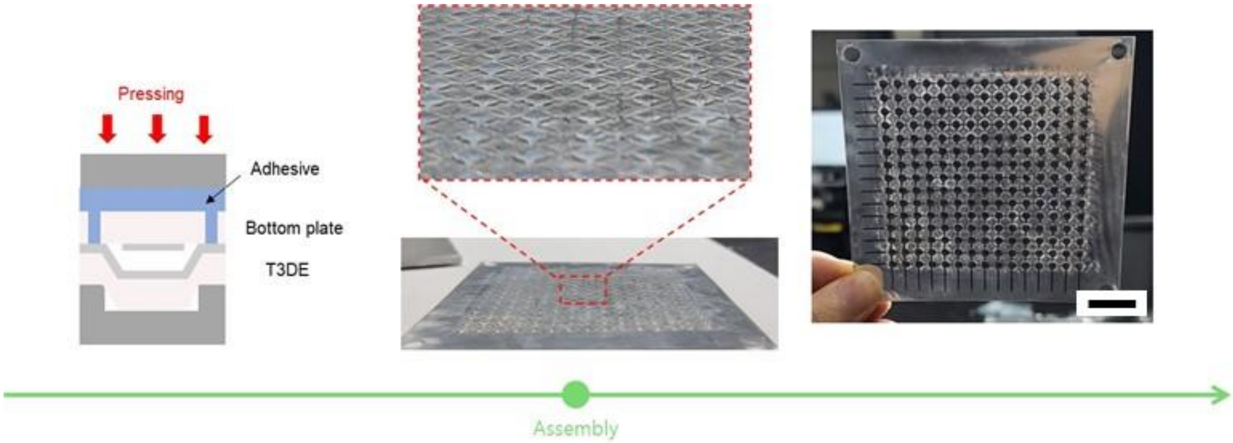

Fig. S17. Assembly of the T3DE and bottom plate. The T3DE is attached to the bottom plate using adhesive, with pressing and heating at 60°C. Proper alignment of the T3DE and the bottom plate is ensured using alignment marks. Various adhesives can be used for this process, with thermal bonding employed in this instance. Scale bar: 20 mm. Photo Credit: Jungrak Choi, ETRI.

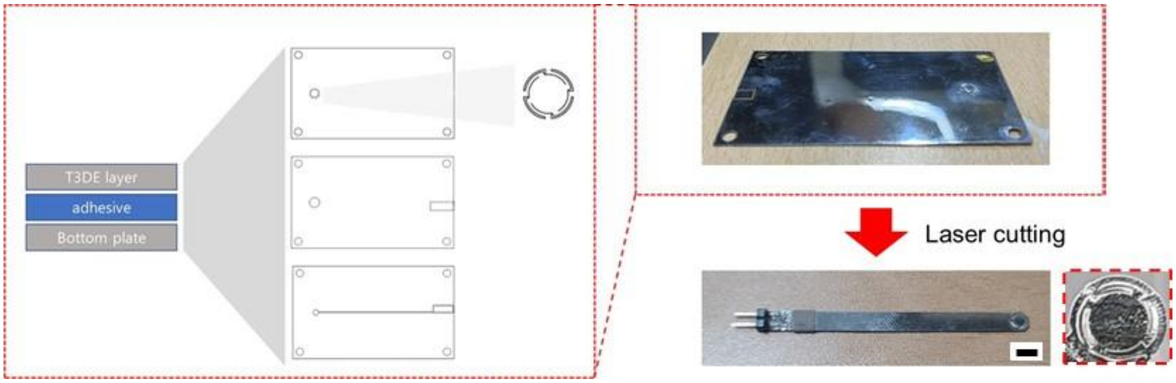

Fig. S18. Fabrication of a single-channel miniature pressure sensor. The T3DE layer, adhesive layer, and bottom plate are patterned using a laser. These layers are then aligned and pressed using an alignment-marked mold. Finally, the sensor's outline is laser-cut, and connectors are attached to complete the assembly. Scale bar: 5 mm. Photo Credit: Jungrak Choi, ETRI.

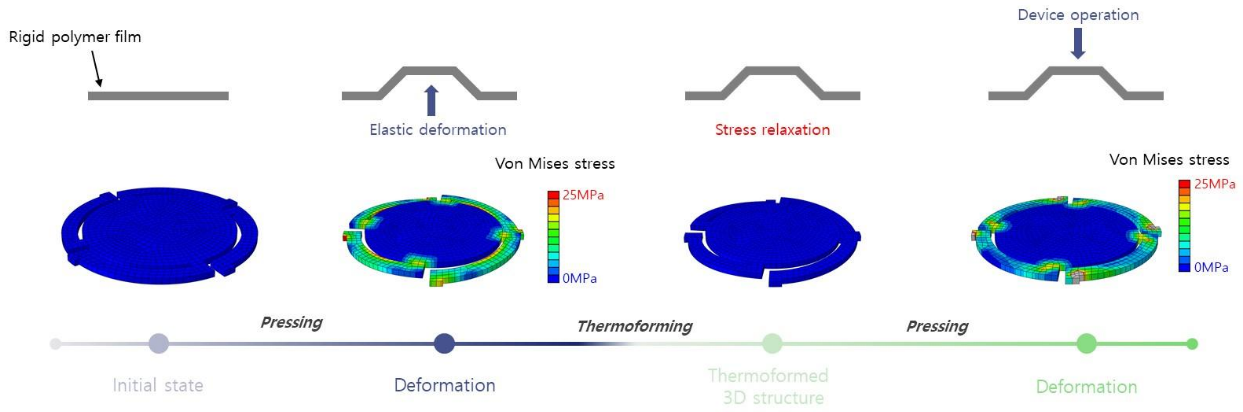

Fig. S19. Simulation process illustrating the stress distribution of the T3DE structure during the thermoforming process. The simulation focuses on how much deformation the structure can undergo without damaging the electrodes. Additionally, the figure includes a schematic of the device operation after thermoforming, showing the stress distribution under external loading conditions.

Table S1. Structural Parameters of T3DE Sensors.

Sensor type	Length (mm)	Number of legs	Width (mm)	Film thickness (mm)	Electrode layer thickness (nm)
1MPa-T3DE	0.7	16	0.3	0.1	200
80kPa-T3DE	1.1	4	0.3	0.1	200
5kPa-T3DE	4.2	4	0.3	0.1	200
300Pa-T3DE	5.4	2	0.3	0.1	200
30Pa-T3DE	9.1	1	0.3	0.1	200

Table S2. Comparison of state-of-the-art capacitive pressure sensors.

Paper	Mechanism	Pressure Range (kPa)	Sensitivity (kPa^{-1})	Hysteresis (%)	Creep error (%)
Ha et al. (*20*)	Capacitive (Porous Nanocomposite Hybrid Response)	0 - 50	3.13 (0-1 kPa), 0.43 (30-50 kPa)	~ 4	-
Huang et al. (*21*)	Capacitive & Transistor-based (Microstructured Air-Gap)	0 – 5	44 (0-5 kPa), Peak 770	-	-
Lee et al. (*22*)	Capacitive (Gallium Microgranules in Elastomer)	0 – 5 (Soft mode)<br>0 – 1000 (Rigid mode)	~ 15 (Soft Mode)<br>~ 4 (Rigid Mode)	~ 3 (Soft Mode)<br>~ 10 (Rigid Mode)	-
Ruth et al. (*25*)	Capacitive (Pyramidal Microstructures)	0 – 40kpa<br>0 – 100kpa<br>0 – 500kpa	Up to ~ 1	-	-
Ruth et al. (*26*)	Capacitive (Tunable Microstructure & Material Properties)	0 – 40kpa<br>0 – 100kpa<br>0 – 500kpa	Up to ~ 1	-	-
Cheng et al. (33)	Capacitive (Hierarchical Microstructure)	0 - 100	3.73	4.42	-
Shao et al. (*34*)	Capacitive (Thermally Expandable Microspheres)	0.01 - 4500	0.201 (under 50 kPa), 3×10^{-4} (MPa range)	~ 12	-
Li et al. (*35*)	Capacitive (Microstructured Electrode)	0 - 1	0.201 (under 50 Pa), 3.3 (~ kPa)	11	-
Shi et al. (*36*)	Capacitive (Screen-Printed Soft Sensor)	-60 to 20<br>-20 to 0<br>0 to 10<br>10 to 20	Up to ~ 1	-	-
Kim et al. (*37*)	Capacitive (Wrinkled Au Electrodes)	0 - 10	0.148	~ 1	-
**Our Work**	**Capacitive (Thermoforming based 3D structure)**	**0 – 0.03<br>0 – 0.3<br>0 – 60<br>0 – 200<br>0 – 400<br>0 - 1000**	**5884<br>~ 1000<br>~ 100<br>~10<br>~ 0.1<br>~ 0.01**	**< 0.5**	**< 1**

Supplementary Videos:

- **Movie S1 (.MP4 format). Tactile Interactions in AR Environment: Pressure Response Visualization.**
- **Movie S2 (.MP4 format). Surgery Stability Training System: Real-Time Sensor Feedback for Excision Stability.**
